
JOURNAL INFORMATION
==============================
NLM Title Abbreviation: Tob Induc Dis
Journal ID: Tob Induc Dis
NLM Journal ID: 101201591
Journal ID: TID

Tobacco Induced Diseases

ISSN: 2070-7266
EISSN: 1617-9625
Publisher: The International Society for the Prevention of Tobacco Induced Diseases

ARTICLE INFORMATION
==============================
PMCID: PMC7083240
PMID: 32206052
DOI: 10.18332/tid/119324
Article ID: 20
Article version: 1
Subjects: Editorial

COVID-19 and smoking: A systematic review of the evidence

Vardavas Constantine I. 1 2
Nikitara Katerina 2
1 Department of Oral Health Policy and Epidemiology, Harvard School of Dental Medicine, Boston, United States
2 School of Medicine, University of Crete, Heraklion, Greece
CORRESPONDENCE TO Constantine I. Vardavas. Department of Oral Health Policy and Epidemiology, Harvard School of Dental Medicine, Boston, United States. E-mail: vardavas@hsdm.harvard.edu
Electronic publication date: 2020 Mar 20
Publication date: 2020
Volume: 18
Electronic Location ID: 20
Received 2020 Mar 19; Accepted 2020 Mar 20
Copyright: © 2020 Vardavas C.I. and Nikitara K
Copyright year: 2020
License: This is an Open Access article distributed under the terms of the Creative Commons Attribution 4.0 International License.
License URL: https://creativecommons.org/licenses/by/4.0/

Keywords: tobacco, smoking, COVID-19, coronavirus

==============================
COVID-19 is a coronavirus outbreak that initially appeared in Wuhan, Hubei Province, China, in December 2019, but it has already evolved into a pandemic spreading rapidly worldwide1,2. As of 18 March 2020, a total number of 194909 cases of COVID-19 have been reported, including 7876 deaths, the majority of which have been reported in China (3242) and Italy (2505)3.

However, as the pandemic is still unfortunately under progression, there are limited data with regard to the clinical characteristics of the patients as well as to their prognostic factors4. Smoking, to date, has been assumed to be possibly associated with adverse disease prognosis, as extensive evidence has highlighted the negative impact of tobacco use on lung health and its causal association with a plethora of respiratory diseases5. Smoking is also detrimental to the immune system and its responsiveness to infections, making smokers more vulnerable to infectious diseases6. Previous studies have shown that smokers are twice more likely than non-smokers to contract influenza and have more severe symptoms, while smokers were also noted to have higher mortality in the previous MERS-CoV outbreak7,8.

Given the gap in the evidence, we conducted a systematic review of studies on COVID-19 that included information on patients’ smoking status to evaluate the association between smoking and COVID-19 outcomes including the severity of the disease, the need for mechanical ventilation, the need for intensive care unit (ICU) hospitalization and death.

The literature search was conducted on 17 March 2020, using two databases (PubMed, ScienceDirect), with the search terms: [‘smoking’ OR ‘tobacco’ OR ‘risk factors’ OR ‘smoker*’] AND [‘COVID-19’ OR ‘COVID 19’ OR ‘novel coronavirus’ OR ‘sars cov-2’ OR ‘sars cov 2’] and included studies published in 2019 and 2020. Further inclusion criteria were that the studies were in English and referred to humans. We also searched the reference lists of the studies included. A total of 71 studies were retrieved through the search, of which 66 were excluded after full-text screening, leaving five studies that were included. All of the studies were conducted in China, four in Wuhan and one across provinces in mainland China. The populations in all studies were patients with COVID-19, and the sample size ranged from 41 to 1099 patients. With regard to the study design, retrospective and prospective methods were used, and the timeframe of all five studies covered the first two months of the COVID-19 pandemic (December 2019, January 2020).

Specifically, Zhou et al.9 studied the epidemiological characteristics of 191 individuals infected with COVID-19, without, however, reporting in more detail the mortality risk factors and the clinical outcomes of the disease. Among the 191 patients, there were 54 deaths, while 137 survived. Among those that died, 9% were current smokers compared to 4% among those that survived, with no statistically significant difference between the smoking rates of survivors and non-survivors (p=0.21) with regard to mortality from COVID-19.

Similarly, Zhang et al.10 presented clinical characteristics of 140 patients with COVID-19. The results showed that among severe patients (n=58), 3.4% were current smokers and 6.9% were former smokers, in contrast to non-severe patients (n=82) among which 0% were current smokers and 3.7% were former smokers , leading to an OR of 2.23; (95% CI: 0.65–7.63; p=0.2).

Huang et al.11 studied the epidemiological characteristics of COVID-19 among 41 patients. In this study, none of those who needed to be admitted to an ICU (n=13) was a current smoker. In contrast, three patients from the non-ICU group were current smokers, with no statistically significant difference between the two groups of patients (p=0.31), albeit the small sample size of the study.

The largest study population of 1099 patients with COVID-19 was provided by Guan et al.12 from multiple regions of mainland China. Descriptive results on the smoking status of patients were provided for the 1099 patients, of which 173 had severe symptoms, and 926 had non-severe symptoms. Among the patients with severe symptoms, 16.9% were current smokers and 5.2% were former smokers, in contrast to patients with non-severe symptoms where 11.8% were current smokers and 1.3% were former smokers. Additionally, in the group of patients that either needed mechanical ventilation, admission to an ICU or died, 25.5% were current smokers and 7.6% were former smokers. In contrast, in the group of patients that did not have these adverse outcomes, only 11.8% were current smokers and 1.6% were former smokers. No statistical analysis for evaluating the association between the severity of the disease outcome and smoking status was conducted in that study.

Finally, Liu et al.13 found among their population of 78 patients with COVID-19 that the adverse outcome group had a significantly higher proportion of patients with a history of smoking (27.3%) than the group that showed improvement or stabilization (3.0%), with this difference statistically significant at the p=0.018 level. In their multivariate logistic regression analysis, the history of smoking was a risk factor of disease progression (OR=14.28; 95% CI: 1.58–25.00; p= 0.018).

We identified five studies that reported data on the smoking status of patients infected with COVID-19. Notably, in the largest study that assessed severity, there were higher percentages of current and former smokers among patients that needed ICU support, mechanical ventilation or who had died, and a higher percentage of smokers among the severe cases12. However, from their published data we can calculate that the smokers were 1.4 times more likely (RR=1.4, 95% CI: 0.98–2.00) to have severe symptoms of COVID-19 and approximately 2.4 times more likely to be admitted to an ICU, need mechanical ventilation or die compared to non-smokers (RR=2.4, 95% CI: 1.43–4.04).

In conclusion, although further research is warranted as the weight of the evidence increases, with the limited available data, and although the above results are unadjusted for other factors that may impact disease progression, smoking is most likely associated with the negative progression and adverse outcomes of COVID-19.

Table 1 Overview of the five studies included in the systematic review

Title	Setting	Population	Study design and time horizon	Outcomes	Smoking rates by outcome	
Zhou et al.9 (2020)
Clinical course and risk factors for mortality of adult inpatients with COVID-19 in Wuhan, China: a retrospective cohort study	Jinyintan Hospital and Wuhan Pulmonary Hospital, Wuhan, China	All adult inpatients (aged ≥18 years) with laboratory confirmed COVID-19 (191 patients)	Retrospective multicenter cohort study until 31 January 2020	Mortality
54 patients died during hospitalisation and 137 were discharged	Current smokers: n=11 (6%)
Non-survivors: n=5 (9%)
Survivors: n=6 (4%)
(p=0.20)

Current smoker vs non-smoker
Univariate logistic regression
(OR=2.23; 95% CI: 0.65–7.63; p=0.2)	
Zhang et al.10
(2020)
Clinical characteristics of 140 patients infected with SARS-CoV-2 in Wuhan, China	No. 7 Hospital of Wuhan, China	All hospitalised patients clinically diagnosed as ‘viral pneumonia’ based on their clinical symptoms with typical changes in chest radiology (140 patients)	Retrospective 16 January to 3 February 2020	Disease Severity
Non-severe
patients: n=82
Severe patients:
n=58	Disease Severity
Former smokers: n=7
Severe: n=4 (6.9%)
Non-severe: n=3 (3.7%) (p= 0.448)

Current smokers: n=2
Severe: n=2 (3.4%)
Non-severe: n=0 (0%)	
Guan et al.12
(2019)
Clinical Characteristics of Coronavirus Disease 2019 in China	552 hospitals in 30 provinces, autonomous regions, and municipalities in mainland China	Patients with laboratory-confirmed COVID-19 (1099 patients)	Retrospective until 29 January 2020	Severity and admission to an ICU, the use of mechanical ventilation, or death
Non-severe patients: n=926 Severe patients: n=173	By severity

Severe cases
16.9% current smokers
5.2% former smokers
77.9% never smokers

Non-severe cases
11.8% current smokers
1.3% former smokers
86.9% never smokers

By mechanical ventilation, ICU or death

Needed mechanical ventilation, ICU or died
25.8% current smokers
7.6% former smokers
66.7% non-smokers

No mechanical ventilation, ICU or death
11.8% current smokers
1.6% former smokers
86.7% never smokers	
Huang et al.11
(2020)
Clinical features of patients infected with 2019 novel coronavirus in Wuhan, China	A hospital in Wuhan, China	Laboratory-confirmed 2019-nCoV patients in Wuhan (41 patients)	Prospective from 16 December 2019 to 2 January 2020	Mortality
As of 22 January 2020, 28 (68%) of 41 patients were discharged and 6 (15%) patients died	Current smokers: n=3
ICU care: n=0
Non-ICU care: n=3 (11%)

Current smokers in ICU care vs non-ICU care patients (p=0.31)	
Liu et al.13 (2019)
Analysis of factors associated with disease outcomes in hospitalised patients with 2019 novel coronavirus disease	Three tertiary hospitals in Wuhan, China	Patients tested positive for COVID-19 (78 patients)	Retrospective multicentre cohort study from 30 December 2019 to 15 January 2020	Disease progression
11 patients (14.1%) in the progression group 67 patients (85.9%) in the improvement/stabilization group 2 deaths	Negative progression group: 27.3% smokers
In the improvement group: 3% smokers

The negative progression group had a significantly higher proportion of patients with a history of smoking than the improvement/stabilisation group (27.3% vs 3.0%)
Multivariate logistic regression analysis indicated that the history of smoking was a risk factor of disease progression (OR=14.28; 95% CI: 1.58–25.00; p= 0.018)	

CONFLICTS OF INTEREST

The authors declare that they have no competing interests, financial or otherwise, related to the current work. C.I. Vardavas reports that he is the Strategic Development Editor of TID and that there are no conflicts of interest with this current work. The authors have each completed and submitted an ICMJE form for disclosure of potential conflicts of interest.

FUNDING

There was no source of funding for this research.

PROVENANCE AND PEER REVIEW

Commissioned; internally peer-reviewed; fast track article.


REFERENCES

1 Wu JT Leung K Leung GM Nowcasting and forecasting the potential domestic and international spread of the 2019-nCoV outbreak originating in Wuhan, China: a modelling study Lancet 2020 395 10225 689 697 10.1016/S0140-6736(20)30260-9 32014114 PMC7159271
2 Li Q Guan X Wu P Early transmission dynamics in Wuhan, China, of novel coronavirus–infected pneumonia N Engl J Med 2020 10.1056/NEJMoa2001316 PMC7121484 31995857
3 ECDC Situation update worldwide, as of March 19 2020 https://www.ecdc.europa.eu/en/geographical-distribution-2019-ncov-cases. Accessed March 19, 2020
4 Khot WY Nadkar MY The 2019 Novel Coronavirus Outbreak–A Global Threat J Assoc Physicians India 2020 68 3 67 32138488
5 Tonnesen P Marott JL Nordestgaard B Bojesen SE Lange P Secular trends in smoking in relation to prevalent and incident smoking-related disease: A prospective population-based study Tob Induc Dis 2019 17 October 10.18332/tid/112459 PMC6830353 31768164
6 Zhou Z Chen P Peng H Are healthy smokers really healthy? Tob Induc Dis 2016 14 November 10.1186/s12971-016-0101-z PMC5111288 27891067
7 Park JE Jung S Kim A MERS transmission and risk factors: a systematic review BMC Public Health 2018 18 1 574 10.1186/s12889-018-5484-8 29716568 PMC5930778
8 Arcavi L Benowitz NL Cigarette smoking and infection Arch Intern Med 2004 164 20 2206 2216 10.1001/archinte.164.20.2206 15534156
9 Zhou F Yu T Du R Clinical course and risk factors for mortality of adult inpatients with COVID-19 in Wuhan, China: a retrospective cohort study Lancet 2020 10.1016/S0140-6736(20)30566-3 PMC7270627 32171076
10 Zhang JJ Dong X Cao YY Clinical characteristics of 140 patients infected by SARS-CoV-2 in Wuhan, China Allergy 2020 10.1111/all.14238 32077115
11 Huang C Wang Y Li X Clinical features of patients infected with 2019 novel coronavirus in Wuhan, China Lancet 2020 395 10223 497 506 10.1016/S0140-6736(20)30183-5 31986264 PMC7159299
12 Guan WJ Ni ZY Hu Y Clinical characteristics of coronavirus disease 2019 in China N Engl J Med 2020 10.1056/NEJMoa2002032 PMC7092819 32109013
13 Liu W Tao ZW Lei W Analysis of factors associated with disease outcomes in hospitalised patients with 2019 novel coronavirus disease Chin Med J 2020 10.1097/CM9.0000000000000775 PMC7147279 32118640
